# Factors associated with hospitalization and death among TB/HIV co-infected persons in Porto Alegre, Brazil

**DOI:** 10.1371/journal.pone.0209174

**Published:** 2019-01-02

**Authors:** Maíra Rossetto, Évelin Maria Brand, Renata Mendonça Rodrigues, Laura Serrant, Luciana Barcellos Teixeira

**Affiliations:** 1 Department of Medicine, Universidade Federal da Fronteira Sul, Chapecó, Santa Catarina, Brasil; 2 Department of Public Health, Universidade Federal do Rio Grande do Sul, Porto Alegre, Rio Grande do Sul, Brasil; 3 Nursing School, Universidade do Estado de Santa Catarina, Chapecó, Santa Catarina, Brasil; 4 Faculty of Health and Wellbeing, Sheffield Hallam University, South Yorkshire, England, United Kingdom; University of New South Wales, AUSTRALIA

## Abstract

In locations with a high rate of tuberculosis (TB) and HIV infection, there are a number of strategies to prevent negative outcomes such as opportunistic infections, hospitalizations and death, and this article investigates risk factors for the occurrence of hospitalization and death in cases of TB/HIV co-infection in the south of Brazil. The data are taken from a population-based retrospective cohort study on cases of TB/HIV co-infection from 2009 to 2013 in Porto Alegre, Brazil. Sociodemographic, epidemiological and clinical variables were analyzed. Relative risk (RR) estimates for hospitalization and death were determined by regression models. There were 2,419 co-infection cases, of which 1,527 (63.1%) corresponded to hospitalizations, and 662 (27.4%) to death. The occurrence of hospitalization was associated with ≤ 7 years of schooling (RR = 3.47, 95%CI: 1.97–6.29), 8–11 years of schooling (RR = 2.56, 95%CI: 1.44–4.69), place of origin—district health authorities Northwest/Humaitá/Navegantes/Ilhas (RR = 2.01, 95%CI: 1.44–2.82), type of entry into the surveillance system as in cases of reentry after withdrawal (RR = 1,35, 95%CI: 1.07–1.70), closure in surveillance as in withdrawal of treatment (RR = 1.47, 95%CI: 1.18–1.83) and multidrug-resistant tuberculosis (RR = 3.94, 95%CI: 1.97–8.81). The occurrence of death was associated with age (RR = 1.07, 95%CI: 1,01–1,14), ≤ 7 years of schooling (RR = 3.94, 95%CI: 2.26–7.09), 8–11 years of schooling (RR = 2.84, 95%CI: 1.61–5.16), place of origin—district health authorities Baltazar (RR = 2.05, 95%CI: 1.48–2.86), type of entry in the surveillance system as cases of re-entry after withdrawal (RR = 1.53, 95%CI: 1.22–1.91), relapse (RR = 1.33, 95%CI: 1.03–1.73). The occurrence of hospitalizations and deaths is high among co-infected patients. Our estimation approach is important in order to identify, from the surveillance data, the risk factors for hospitalization and death in co-infected patients, so that they may receive more attention from the Brazilian national healthcare system.

## Introduction

Tuberculosis and human immunodeficiency virus (TB/HIV) co-infection is one of the most complex clinical pictures in the field of public health [1–2]. The double burden of diseases makes adherence to treatments difficult, a fact that is shown by high default rates, which contribute to multidrug resistance [3–5]. Likewise, the social vulnerability of the patients may lead to an increase in hospitalization rates and mortality [5–8]. Risk factors for hospitalization and death in locations with a high incidence of co-infection such as Brazil have not been sufficiently investigated.

Studies point out that the HIV/AIDS epidemic has contributed to the growth of the incidence of tuberculosis [5,9]. People living with HIV/AIDS have a 3 to 15% annual risk of reactivating latent tuberculosis infection, while the risk for the general population is only 0.1% [10]. Considering the period from 2000 to 2016, tuberculosis remains among the ten leading causes of death in the world [11]. In Brazil, the estimated mortality rate due to co-infection was 0.9 per 100,000 inhabitants in 2016, which is higher than the rate in the Americas. Tuberculosis was one of the world’s leading causes of death for people living with HIV in 2017, when deaths related to HIV reached approximately 1.4 million, 400,000 of them being caused by tuberculosis [1].

In Brazil, the southern region has the highest percentage of co-infection in the country (18.2%), almost twice the national average [12,13]. Porto Alegre is the state capital with the highest incidence of HIV/AIDS and the second highest incidence of tuberculosis. The city has the largest proportion of co-infected people in Brazil, with 25% of the tuberculosis cases occurring in people living with HIV. These indicators show the striking impact of the disease in Porto Alegre as compared to other Brazilian cities [12,13].

Many strategies have been discussed to prevent hospitalization and death in locations pointed out by the World Health Organization as having a great burden of tuberculosis and HIV infection [1,2,13–15]. In this regard, Brazil is considered a pioneer since its adoption in 1996 of antiretroviral therapy for all people living with HIV, regardless of CD4 count [16]. Moreover, the Global Fund to Fight AIDS, Tuberculosis and Malaria has been increasing its actions to reduce TB/HIV co-infection by expanding the Directly Observed Treatment Short-Course (DOTS) strategy in large urban centers such as Porto Alegre [17] with a high tuberculosis burden [1,2].

Brazil is among the top 20 countries worldwide with the highest incidence of TB/HIV co-infection, with 13 cases per 100,000 inhabitants. The most affected countries are on the African continent with rates of up to 72 cases per 100,000 inhabitants [1]. Studies show that the occurrence of hospitalization and death in co-infected patients is considerably high [3,5,7,8,18–20]. In Ethiopia, for example, mortality in patients with co-infection reaches 20.2% [8]. In Cameroon, a cohort recruited during eight years showed a proportion of almost 30% of deaths in hospitalized co-infected patients [18]. This situation implies high social and financial costs for public health systems, especially in Brazil, where the system is universal and totally free [1]. Given the importance and scale of the problem, a study was designed to analyze the risk factors for hospitalization and death in cases of TB/HIV co-infection in Porto Alegre.

## Materials and methods

This is a population-based, retrospective cohort study that consisted of the analysis of all cases of pulmonary tuberculosis and HIV co-infection among residents of the city of Porto Alegre from 2009 to 2013.

We used the following sources of information: cases of tuberculosis and AIDS registered in the Brazilian Information System for Notifiable Diseases (SINAN) over a period of five years (2009–2013); hospitalizations recorded in the Hospital Information System (SIH) over a period of three years after the diagnosis of each patient; and deaths recorded in the Mortality Information System (SIM) over a period of two years. This follow-up period was defined in the methodology of the study, taking into account the time to update the national systems used (SIM and SIH). The hospitalization data were collected until 2016, although the cohort was from 2009 to 2013, aiming at the same follow-up period for all. For example, patients who entered the cohort in 2013 were followed up until 2016 (with a 3-year follow-up). The mortality system is updated every 2 years, so mortality data for 2016 are being entered into the system in 2018. In order to be able to complete 3 years of following up the mortality of the new patients in 2013, we would have to access data for 2016, which will be made available in the system this year, 2018. Thus, in order to achieve a 2-year follow-up on mortality for patients who entered in 2013 (follow-up until 2015), the data until December 2017 were accessed. These data were available on the national systems at the end of the study. Only hospitalizations and deaths related to TB/HIV were considered. Tuberculosis/HIV/AIDS co-infection was defined as every case reported as pulmonary tuberculosis in SINAN-TB that had a confirmed AIDS or HIV positive test. Subsequently, all cases with tuberculosis that had an anti-HIV test with negative or in progress results were checked in the SINAN-AIDS database to confirm co-infection.

After a single database using SINAN data was created, SIH and SIM were respectively checked for hospitalizations and deaths. The linkage between SINAN, SIH and SIM databases included the individual's full name, date of birth and mother's name. The data available in SINAN, which is a mandatory notification system, is registered at the time of TB/HIV diagnosis and used for the investigation of hospitalizations and mortality.

As far as the organization of health care services and resources is concerned, the city of Porto Alegre is divided into eight district health authorities (DHAs): Centro (CEN), Baltazar (NEB), East/Northeast (LENO), Gloria/Cruzeiro/Cristal (GCC), South/Center South (SCS), Parthenon/Lomba do Pinheiro (PLP), Restinga/South End (RES), Northwest/Humaitá/Navegantes/Ilhas (NHNI). This geographical division was used to analyze the place of origin of the cases. The reference category for the RR analysis was defined after the proportions of the hospitalization and death events were assessed at the respective locations. We chose the location with the lowest hospitalization and death rates as the reference category for this variable.

Sociodemographic and epidemiological variables were also investigated. The former included place of origin (DHA), age, gender, race/ethnicity and education; the latter, type of entry in SINAN, prescription of directly observed therapy (DOT) and status at completion. The type of entry was classified into new case (never treated), relapse (patient cured and infected once again by tuberculosis), return after default (patient who interrupted the treatment and is about to resume it) or transfer (when the individual was being treated at another health facility and was transferred). The *new case* category was defined as the reference category for analysis. The variable *status at completion* refers to what happened after the tuberculosis treatment was monitored by the national surveillance system, that is: cure, default, transference, death or multiresistance. In this variable, the category *cure* was defined as the reference category.

We performed the analysis using two softwares: *Statistical Package for the Social Sciences (SPSS)*, version 18.0, and *R*, version 3.2.0. The chi-square test of Person and the Fisher's exact test were used to compare groups in relation to outcomes, whereas the T-student test was used for independent samples. Measures of association for each category of the exploratory variables were estimated through regression models, with reference to the lowest expected risk category. Predictor variables were included in the regression models based on theoretical knowledge of the outcomes through univariate models. For the multi-variable model, those variables with clinical importance or with a p-value less than 0.25 were included. The multivariable model estimated the associations for each exploratory variable in a regression model that simultaneously included all variables, in order to examine the independent effect of each exploratory variable on the outcome. For crude and adjusted measures of association, the Wald test was used. The significance of 5% was adopted to determine whether the variable was associated with the outcome in the multi-variable model. RR values, p value and 95% confidence interval values were used for the modeling of each outcome,

This study complied with the guidelines of the Resolution 466/2012 of the Brazilian National Health Council. The research project was approved by the Ethics and Research Committee of the Federal University of Rio Grande do Sul (UFRGS) and by the Ethics Committee of the Municipal Government of Porto Alegre, opinions 952907 and 939250, respectively. Since this is a linkage study of national databases, patients needed to be identified for the linkage procedure to succeed. This was informed in the research protocol to the ethics committee, which approved the study waiving the need for a term of consent.

## Results

There were 2,419 cases of TB/HIV co-infection in Porto Alegre, of which 63.1% were hospitalised and 27.4% died in the period studied.

Table 1 shows the sociodemographic, epidemiological and clinical characteristics of the co-infected patients in Porto Alegre, according to the occurrences of hospitalization and death. The factors significantly associated with hospitalization were: DHA, schooling, entry situation and implementation of DOT. The factors significantly associated with death were schooling, age, DOT recommendation and implementation of DOT.

**Table 1:** Socio-demographic, epidemiological and clinical characteristics of TB/HIV co-infected persons according to the occurrence of hospitalization and death in Porto Alegre, Brazil, from 2009 to 2013.

Characteristics	Total	Hospitalization	p-value	Death	p-value
DHA^*^			<0.001^c^		0.462^c^
CEN	430 (17.8%)	274 (63.7%)		111 (25.8%)	
NHNI	159 (6.6%)	99 (62.3%)		48 (30.2%)	
NEB	264 (10.9%)	186 (70.5%)		74 (28%)	
LENO	380 (15.7%)	239 (62.9%)		106 (28%)	
GCC	286 (11.8%)	193 (67.5%)		82 (28,7%)	
SCS	170 (7%)	117 (68.8%)		50 (29.4%)	
PLP	557 (23%)	309 (55.5%)		135 (24.3%)	
RES	171 (7.1%)	108 (63.2%)		55 (32.4%)	
**Race/ethnicity**			0.097^c^		0.978^a^
White	1357 (56.3%)	838 (61.8%)		371 (27.4%)	
Nonwhite	1054 (43.7%)	686 (65.2%)		288 (27.4%)	
**Gender**			0.965^a^		0.564^a^
Male	1588 (65.6%)	1003 (63.2%)		441 (27.8%)	
Female	831 (34.4%)	524 (63.1%)		221 (26.7%)	
**Years of education completed**			<0.001^c^		0.013^c^
≤7 years	1548 (69.2%)	1027 (66.3%)		433 (28%)	
From 8 to 11 years	629 (28.1%)	366 (58.2%)		151 (24.1%)	
≥12 years	59 (2.6%)	19 (32.2%)		8 (13.6%)	
**Age**	38 ± 9.91	37.88 ± 9.93	0.422^b^	39.93 ±10.7	< 0.001^b^
**Type of entry**			<0.001^c^		0.068^c^
New case	1389 (57.4%)	825 (59.4%)		378 (27.3%)	
Relapse	351 (14.5%)	233 (66.4%)		100 (28.5%)	
Return after default	622 (25.7%)	438 (70.4%)		177 (28.5%)	
Transfer	57 (2.4%)	31 (54.4%)		7 (12.3%)	
Prescription of DOT^**^			0.532^a^		<0.001^a^
Yes	627 (26%)	389 (62%)		122 (19.5%)	
No	1785 (74%)	1133 (63.5%)		538 (30.2%)	
DOT^**^ **completed**			0.037^a^		<0.001^a^
Yes	406 (16.9%)	275 (67.7%)		74 (18.3%)	
No	1994 (83.1%)	1240 (62.2%)		581 (29.2%)	
**Total**	**2.419 (100%)**	**1.527 (63.1%)**		**662 (27.4%)**	

^a^p-value associated with Fisher’s exact test

^b^p-value associated with the T-test for independent samples

^c^Test of homogeneity of proportions based on Pearson's chi-square test

*District Health Authorities (DHA): Centro (CEN), Baltazar (NEB), East/Northeast (LENO), Gloria/Cruzeiro/Cristal (GCC), South/Center South (SCS), Parthenon/Lomba do Pinheiro (PLP), Restinga/South End (RES), Northwest/Humaitá/Navegantes/Ilhas (NHNI)

**DOT = Directly observed therapy.

Table 2 presents the risk factors for the occurrence of hospitalization in cases of TB/HIV co-infection in Porto Alegre. In the crude regression model, the linearity of the variable *year* was verified, and it was observed that, for each year, the risk of hospitalization increased 7% (RR = 1.07; CI 95%: 1.01–1.14). The variable *education* was predictive for the occurrence of hospitalization (p <0.001). As to the place of origin, the proportion of hospitalizations was lower in PLP, and therefore this was considered a reference category. Compared to this category, the other DHAs showed a positive association with the outcome. In terms of the type of entry registered in SINAN, relapse cases had 1.36 times more risk of hospitalization (RR = 1.36; CI 95%: 1.06–1.74); the cases of return after default, 1.65 times more risk (RR = 1.65, CI 95%: 1.34–2.02); and transfer was not significant. Considering the completion status registered in SINAN, default cases had 1.65 times more risk of hospitalization (RR = 1.65; CI 95%: 1.36–2.01) and multidrug-resistant TB (MDR), 4.89 times more risk (RR = 4.89; CI 95%: 2.52–10.8). Transfer was not associated with the outcome.

**Table 2:** Risk factors for hospitalization in cases of TB/ HIV co-infection in Porto Alegre, from 2009 to 2013.

Characteristics	Crude RR (CI 95%)^a^	Adjusted RR (CI 95%)^b^
**Year**	1.07 (1.01–1.14)*	1.06 (0.99–1.13)
**Gender**	**p = 0.937**	**p = 0.434**
Male	Reference	Reference
Female	1.01 (0,85–1.2)	0.93 (0.76–1.12)
**Age**	1.00 (0.99–1)**	1 (0.99–1.01)
**Years of education completed**	**p<0.001**	**p<0.001**
≤7	4.14 (2.41–7,38)	3.47 (1.97–6.29)
8–11	2.93 (1.68–5.28)	2.56 (1.44–4.69)
≥ 12	Reference	Reference
**Race ∕ethnicity (self-declared)**	**p = 0.120**	**p = 0.475**
White	Reference	Reference
Others	1.14 (0.97–1.35)	1.07 (0.89–1.29)
**DHA**^**c**^	**p = 0.001**	**p<0.001**
CEN	1.40 (1.08–1.81)	1.4 (1.05–1.87)
GCC	1.67 (1.24–2.26)	1.42 (1.03–1.96)
LENO	1.35 (1.04–1.77)	1.23 (0.91–1.65)
NHNI	1.35 (0.94–1.95)	2.01 (1.44–2.82)
NEB	1.93 (1.42–2.66)	1.43 (0.97–2.13)
PLP	Reference	Reference
RES	1.38 (0.97–1.97)	1.31 (0.9–1.92)
SCS	1.78 (1.24–2.57)	1.95 (1.32–2.92)
**Type of entry**	**p<0.001**	**p<0.001**
New case	Reference	Reference
Relapse	1.36 (1.06–1.74)	1.26 (0.97–1.65)
Return after default	1.65 (1.34–2.02)	1.35 (1.07–1.7)
Transfer	0.71 (0.41–1.24)	0.67 (0.35–1.25)
**Status at completion**	**p<0.001**	**p<0.001**
Cure	Reference	Reference
Default	1.65 (1.36–2.01)	1.47 (1.18–1.83)
MDR-TB^d^	4.89 (2.52–10.8)	3.94 (1.97–8.81)
Transfer	0.71 (0.45–1.11)	0.74 (0.46–1.19)
**Prescription of DOT**^**e**^	**p = 0.478**	**p = 0.197**
Yes	Reference	Reference
No	1.07 (0.89–1.29)	0.87 (0.7–1.08)

^a^univariate models

^b^multi-variable model by the logistic regression method

*p-value <0,05

**p-value = 0,455

^c^District Health Authorities (DHA): Centro (CEN), Baltazar (NEB), East/Northeast (LENO), Gloria/Cruzeiro/Cristal (GCC), South/Center South (SCS), Parthenon/Lomba do Pinheiro (PLP), Restinga/South End (RES), Northwest/Humaitá/Navegantes/Ilhas (NHNI)

^d^MDR-TB = Multidrug-resistant TB

^e^DOT = Directly observed therapy.

In the regression model adjusted for the hospitalization outcome (Table 2), the variables showing a positive association with the outcome were as follows: education less than or equal to 7 years (RR = 3.47, CI 95%: 1.97–6.29); education from 8 to 11 years (RR = 2.56, CI 95%: 1.44–4.69); DHA CEN (RR = 1.4, 95% CI: 1.05–1.87); DHA GCC (RR = 1.42, CI 95%: 1.03–1.96); entry in SINAN, where return after default presented 1.35 times more risk of hospitalization (RR = 1.35, CI 95%: 1.07–1.7); and status at completion, whose categories default (RR = 1.47, CI 95%: 1.18–1.83), death (RR = 1.94, CI 95%: 1.5–2.51) and MDR-TB (RR = 3.94, CI 95%: 1.97–8.81) presented positive association with the outcome.

Table 3 presents the risk factors for death in cases of TB/HIV co-infection in Porto Alegre. In the crude regression model for the mortality outcome, the linearity of the *year* and *age* variables were verified, and it was observed that, for each year of the analyzed period, the risk of death decreased by 10% (RR = 0.91, CI 95% 0.85–0.97). Meanwhile, for every additional year in the age of notification, the risk of death increased by 3% (RR = 1.03, CI 95%: 1.01–1.04). Nevertheless, these variables were not associated with the outcome in the multivariable model. Level of education proved to be associated with mortality. As to the place of origin, the proportion of deaths was lower in PLP, and therefore this was considered a reference category. Concerning SINAN type of entry, *new case* was used as a reference category, and there was no statistical differences in the other categories, except for transfer, which showed a negative association with the mortality outcome (RR = 0.41, CI 95%: 0.17–0.85). Cases without prescription of DOT showed 1.77 times more risk of death than those with it (RR = 1.77, CI 95%: 1.42–2.22).

**Table 3:** Risk factors for mortality in cases of TB/HIV co-infection in Porto Alegre, from 2009 to 2013.

Characteristics	Crude RR (CI 95%)^a^	Adjusted RR (CI 95%)^b^
**Year**	0.91 (0.85–0.97)**	1.07 (1.01–1.14)*
**Gender**	**p = 0.564**	**p = 0.675**
Male	Reference	Reference
Female	1.06 (0.87–1.28)	0.96 (0.79–1.16)
**Age**	1.03 (1.01–1.04)***	1 (0.99–1.01)
**Years of Education**	**p = 0.008**	**p<0.001**
≤7	2.49 (1.24–5.71)	3.94 (2.26–7.09)
8–11	2.02 (0.99–4.69)	2.84 (1.61–5.16)
≥ 12	Reference	Reference
**Race ∕ethnicity (self-declared)**	**p = 0.996**	**p = 0.477**
White	Reference	Reference
Others	1.00 (0.83–1.,2)	1.07 (0.89–1.29)
**DHA**^**c**^	**p = 0.501**	**p<0.001**
CEN	1.09 (0.81–1.46)	1.54 (1.17–2.05)
GCC	1.25 (0.9–1.72)	1.59 (1.16–2.18)
LENO	1.2 (0.89–1.61)	1.37 (1.03–1.84)
NHNI	1.35 (0.91–1.99)	1.53 (1.04–2.26)
NEB	1.22 (0.87–1.7)	2.05 (1.48–2.86)
PLP	Reference	Reference
RES	1.47 (1.01–2.13)	1.39 (0.96–2.03)
SCS	1.29 (0.88–1.89)	2.04 (1.39–3.05)
**Type of entry**	**p = 0.07**	**p<0.001**
New case	Reference	Reference
Relapse	1.08 (0.83–1.39)	1.33 (1.03–1.73)
Return after default	1.08 (0.87–1.33)	1.53 (1.22–1.91)
Transfer	0.41 (0.17–0.85)	0.66 (0.36–1.23)
**Prescription of DOT**^**d**^	**p<0.001**	**p = 0.085**
Yes	Reference	Reference
No	1.77 (1.42–2.22)	0.83 (0.68–1.03)

^a^univariate models

^b^multi-variable model by the logistic regression method

*p-value <0.05

**p-value <0.01

***p-value <0.001

^c^District Health Authorities (DHA): Centro (CEN), Baltazar (NEB), East/Northeast (LENO), Gloria/Cruzeiro/Cristal (GCC), South/Center South (SCS), Parthenon/Lomba do Pinheiro (PLP), Restinga/South End (RES), Northwest/Humaitá/Navegantes/Ilhas (NHNI)

^d^DOT = Directly observed therapy.

In the regression model adjusted for the mortality outcome (Table 3), the following variables showed association with the outcome: education, in which categories up to 7 years (RR = 3.94, CI 95%: 2.26–7.09) and 8 to 11 years (RR = 2.84, CI 95%: 1.61–5.16) presented risk of mortality; place of origin, whose categories CEN (RR = 1.54, CI 95%: 1.17–2.05), GCC (RR = 1.59, CI 95%: 1.16–2.18), LENO (RR = 1.37, CI 95%: 1.03–1.84), NHNI (RR = 1.53, CI 95%: 1.04–2.26), NEB (RR = 2.05, CI 95% 1.48–2.86) and SCS (RR = 2.04, CI 95%: 1.39–3.05) presented risk of mortality; and SINAN type of entry, where category return after default presented 1.53 times more risk of mortality than the reference category (RR = 1.53, CI 95%: 1.22–1.91).

## Discussion

This study shows that more than 60% of TB/HIV co-infection cases resulted in at least one hospitalization and that nearly 30% of the patients died. Our mortality data over a five-year period is similar to that of an eight-year cohort conducted in Cameroon with hospitalized patients, where 29% died [18]. In another study carried out in Ethiopia, 7.6% out of 158 co-infected patients under outpatient treatment died [19]. The vulnerability of co-infected individuals to opportunistic infections may explain the high occurrence of such outcomes [3,19,21], especially in a location with high default rates [3,22,23] such as Porto Alegre [12,13]. Against this background, the importance of the adherence to the tuberculosis treatment [24–26] has been highlighted in health care policies, since it prevents multidrug resistance and eventually leads to cure. Adherence to treatment also prevents new opportunistic infections in people living with HIV [1].

Regarding sociodemographic characteristics, this study confirmed the results of previous research [27,28]. The profile of the co-infected individual is a white males with a low level of education and a mean age of 38 ± 9,91 years. The risk of death was higher for every additional year of age at the time of the notification of co-infection (p<0.05), and this may indicate the probability of death due to late diagnosis in a scenario with a growing number of cases of co-infection [29]. The effect of calendar time for hospitalisation was very close to significance. An explanatory hypothesis for this finding is the fragility of specialized services in ensuring continued care to promote adherence to treatment in a scenario with the highest prevalence of TB and the highest rate of AIDS detection in the country. It may also be related to difficulties in accessing health services, as seen in other studies [30,31], despite the free availability of medication for tuberculosis and AIDS in Brazil [2]. Another weakness in the Brazilian health system is the difficulty of identifying the initial symptoms of TB, which makes it impossible to diagnose the disease in patients living with HIV [30].

Co-infection has a great social impact. Most of the patients are economically active and generally provide for their families, contributing to the social and economic development of their communities [18,32]. The social costs of the illness result also from the need for long-term follow-up and treatment [33]. Failure in access and adherence, added to difficulties related to the treatment, often culminate in neglect, relapses, frequent hospitalizations and high mortality rates [29,34,35].

In the sample studied, a low level of education was identified as a risk factor for hospitalization (p<0.001) and death (p<0.001). The proportion of co-infected patients with a low level of education was higher than that found in other Brazilian studies [36,37], pointing to a different profile in the city of Porto Alegre. In the national systems used in this study, income information was not available, and so the level of education was used as a proxy for socioeconomic status and was significantly associated with the outcomes. In fact, it was observed that the lower the level of education, the higher the risk both for hospitalization and mortality. Studies have suggested that individuals with poor education may postpone the demand for health services or find barriers to accessing it. Consequently, they find it difficult to receive diagnosis and treatment [28,38], a process leading to hospitalization and death.

Co-infection cases with the highest risk of hospitalization (p<0.001) and death (p<0.001) belonged to DHAs of extreme social vulnerability [39,40]. Other studies also related the highest occurrence of tuberculosis, HIV, and death due to tuberculosis to locations distinguished by their severe poverty [23,41,42]. People with lower income and education are more vulnerable to therapeutic failure and poor adherence to treatment [9,19]. A recent qualitative study conducted in the same setting [8] showed that social vulnerability is related to hospitalization for tuberculosis. Regarding the type of entry in the surveillance system, cases registered as return after treatment default were 35% more likely to be hospitalized and 53% more likely to die than new cases (p<0.001). Likewise, relapses were 26% more likely to be hospitalized and 33% more likely to die than new cases (p<0.001). With regard to treatment default, it must be noted that adherence to treatment is one of the most important topics in the public policy agenda in view of the difficulty to manage the double burden of diseases [43]. In fact, several studies have emphasized the benefits of the early initiation of treatment for patients recently diagnosed with HIV [18,19,44,45].

The proportion of cases found in the category return after default highlights the need to qualify the current actions concerning adherence to treatment in Brazil. This is all the more important as treatment discontinuation can significantly impact the occurrence of multidrug-resistant tuberculosis [46,47]. The cases of multidrug-resistant tuberculosis presented a risk of hospitalization 3.94 times higher than new cases (p<0.001). Multidrug-resistant tuberculosis has been associated with a higher exposure to opportunistic diseases and increased mortality [20,41,44,48].

In this study, the transfer of the patient to another health care service was not maintained in the multivariable model of the outcomes. Transfer situations usually occur when individuals leave the Rio Grande do Sul state capital of Porto Alegre for towards cities in the hinterland. This fact probably indicates the quality and pro-activity of small city and town health services compared to those of Porto Alegre. Currently, Porto Alegre provides public health services for a population of approximately 1,500,000 residents and is a reference center for the care of more than 3 million people in the municipalities of the metropolitan region.

Directly observed therapy (DOT) [1], although used worldwide [49–51], did not stand out as a therapeutic approach in the cases studied. Although it was indicated for almost 30% of the cases, it was performed only in 16.9%. In spite of this difference, DOT was not associated with the outcomes in the adjusted models. Patients who entered the surveillance system in the category *return after default* presented a higher risk of hospitalization and mortality, showing the direct influence of non-adherence to treatment. These patients benefitted from DOT. However, in Porto Alegre, primary care services have yet not sufficiently incorporated this technology [52]. Our study has certain limitations. It did not include in its analysis information about treatment regimens or CD4 counts, once they were not stored in the accessed databases. We used schooling as a proxy of socioeconomic level as there was no available information on disposable income, and the use of education in place of socio-economic status might fail to properly discriminate between rich and poor. Therefore, further studies on the management and adherence of co-infected patients are strongly recommended. Additional research on hospital admissions can also contribute to the management of co-infected patients in Porto Alegre. This is all the more important as death from tuberculosis is often the last stage of a health-disease process that could well be avoided.

## Supporting information

S1 Dataset Co-infection in Porto Alegre (XLX). (XLS)
