# Correction: Factors associated with hospitalization and death among TB/HIV co-infected persons in Porto Alegre, Brazil

**DOI:** 10.1371/journal.pone.0224230

**Published:** 2019-10-16

**Authors:** The PLOS ONE Staff

## Notice of Republication

An incorrect version of Supporting information file S1 Dataset was published in error. The publisher apologizes for this error. This article was republished on October 8, 2019 to correct for this error. Please download this article again to view the correct version.
